# Zinc Finger Transcription Factor Zbtb16 Coordinates the Response to Energy Deficit in the Mouse Hypothalamus

**DOI:** 10.3389/fnins.2020.592947

**Published:** 2020-12-01

**Authors:** Helia Cheng, Schuyler J. Pablico, Jisu Lee, Ji Suk Chang, Sangho Yu

**Affiliations:** ^1^’Department of Neurobiology of Nutrition and Metabolism, Louisiana State University System, Baton Rouge, LA, United States; ^2^Department of Gene Regulation and Metabolism, Pennington Biomedical Research Center, Louisiana State University System, Baton Rouge, LA, United States

**Keywords:** PLZF, food intake, energy expenditure, energy balance, energy deficit, glucocorticoid

## Abstract

The central nervous system controls feeding behavior and energy expenditure in response to various internal and external stimuli to maintain energy balance. Here we report that the newly identified transcription factor zinc finger and BTB domain containing 16 (Zbtb16) is induced by energy deficit in the paraventricular (PVH) and arcuate (ARC) nuclei of the hypothalamus via glucocorticoid (GC) signaling. In the PVH, *Zbtb16* is expressed in the anterior half of the PVH and co-expressed with many neuronal markers such as corticotropin-releasing hormone (Crh), thyrotropin-releasing hormone (Trh), oxytocin (Oxt), arginine vasopressin (Avp), and nitric oxide synthase 1 (Nos1). Knockdown (KD) of *Zbtb16* in the PVH results in attenuated cold-induced thermogenesis and improved glucose tolerance without affecting food intake. In the meantime, *Zbtb16* is predominantly expressed in agouti-related neuropeptide/neuropeptide Y (Agrp/Npy) neurons in the ARC and its KD in the ARC leads to reduced food intake. We further reveal that chemogenetic stimulation of PVH Zbtb16 neurons increases energy expenditure while that of ARC Zbtb16 neurons increases food intake. Taken together, we conclude that *Zbtb16* is an important mediator that coordinates responses to energy deficit downstream of GCs by contributing to glycemic control through the PVH and feeding behavior regulation through the ARC, and additionally reveal its function in controlling energy expenditure during cold-evoked thermogenesis via the PVH. As a result, we hypothesize that Zbtb16 may be involved in promoting weight regain after weight loss.

## Introduction

The brain constantly monitors the energy status of the body through various neuronal, hormonal, and metabolic signals to maintain energy balance (Gao and Horvath, 2007). The brain achieves this goal by regulating effector pathways controlling feeding behavior and energy expenditure and in doing so, integrates various environmental signals to mount coordinated responses to optimize the survival of an organism. The hypothalamus is an essential brain structure for this critical homeostatic mechanism and involved in multiple aspects of hormonal, behavioral, and autonomic responses that control feeding and energy consumption (Coll and Yeo, 2013). The ARC is the primary sensory region that receives neuronal and humoral signals related to energy balance and probably the most studied site regarding feeding regulation. The ARC contains two intermingled and counteracting neuronal populations that express Agrp/Npy and proopiomelanocortin (Pomc), respectively, and while Agrp/Npy neurons promote feeding, Pomc neurons promote satiety (Andermann and Lowell, 2017). These neurons project to the PVH to exert their function, among other areas (Gao and Horvath, 2007). In addition to feeding behavior control, the PVH regulates various neuroendocrine functions, energy expenditure, and glucose metabolism through multiple functionally distinct neuronal populations (Swanson and Sawchenko, 1980; Sutton et al., 2014; Roh et al., 2016).

We previously identified *zinc finger and BTB domain containing 16* (*Zbtb16*) to be highly upregulated by acute cold exposure in the mouse hypothalamus (unpublished data). Zbtb16, also known as promyelocytic leukemia zinc finger (Plzf), causes human acute promyelocytic leukemia as a fusion protein with retinoic acid receptor α (Borrow et al., 1990; de The et al., 1990). During development, Zbtb16 plays a specific role of balancing the self-renewal and differentiation of stem cells in multiple organs (see (Liu et al., 2016) and references therein) but carries out diverse tissue-specific functions in adult. Nonetheless, the function of Zbtb16 has never been studied in adult brains while its expression and function have been described in developing central nervous system (Avantaggiato et al., 1995; Cook et al., 1995; Gaber et al., 2013; Lin et al., 2019). More careful expression analysis in the brain revealed *Zbtb16* expression in two hypothalamic nuclei, the PVH and the ARC, that are critical for the control of energy balance. Therefore, we set out to uncover the function of hypothalamic Zbtb16 in metabolic regulation in these two nuclei by genetically targeting its expression and modulating neuronal activity.

In the current study, we describe the expression of *Zbtb16* in the mouse brain and the induction of its expression by various energy deficit conditions via GC signaling in the PVH and the ARC. We demonstrate that Zbtb16 in the PVH (Zbtb16^PVH^) is important for cold-induced thermogenic response and glycemic control while Zbtb16 in the ARC (Zbtb16^ARC^) modulates feeding behavior. Therefore, this newly identified transcription factor Zbtb16 may be an important component for responses to energy deficit and contribute to overall energy homeostasis.

## Materials and Methods

### Animals

All experiments were approved by the Institutional Animal Care and Use Committee at Pennington Biomedical Research Center (protocol # 1043). Mice were housed at 22–24°C with a 12:12 light:dark cycle. Laboratory rodent diet (5001, LabDiet) and water were available *ad libitum* unless stated otherwise. For investigation of *Zbtb16* induction by cold, overnight fasting, and 2-deoxy-D-glucose (2DG), and *Zbtb16* KD in the PVH or the ARC, C57BL/6J mice (000664, The Jackson Laboratory, RRID: IMSR_JAX:000664) were used at 2–4 months of age. *Zbtb16-Cre* mice were originally generated by Dr. Albert Bendelac at University of Chicago and obtained through the Jackson Laboratory (024529, The Jackson Laboratory, RRID: IMSR_JAX:024529) (Constantinides et al., 2014). *Npy-GFP* mice (006417, The Jackson Laboratory, RRID: IMSR_JAX:006417) and *single-minded 1 (Sim1)-Cre* mice (006395, The Jackson Laboratory, RRID: IMSR_JAX:006395) were created by Dr. Bradford Lowell at Beth Israel Deaconess Medical Center, Harvard University and obtained through the Jackson Laboratory (Balthasar et al., 2005; van den Pol et al., 2009). *L10-GFP* mice were created by Dr. Andrew McMahon at University of Southern California and obtained from the Jackson Laboratory (024750, The Jackson Laboratory, RRID: IMSR_JAX:024750) (Liu et al., 2014). Both male and female mice were used in all experiments and no sex difference was observed in any measurement.

### Zbtb16 mRNA Expression Analysis

RNA from the whole mouse hypothalamus or microdissected PVH or ARC was purified with TRIzol Reagent (#15596, Thermo Fisher Scientific) and cDNA was synthesized with SuperScript VILO cDNA synthesis kit (#11754050, Thermo Fisher Scientific). *Zbtb16* mRNA expression was analyzed with the TaqMan assay method (Thermo Fisher Scientific) using the 7900HT real-time PCR system (Thermo Fisher Scientific). β-actin was used as a reference gene for relative quantification.

For investigation of hypothalamic *Zbtb16* expression at different temperature, C57BL/6J mice were exposed to cold (4°C), room temperature (RT, 22°C), and warm (35°C) for 3 h. Brains were harvested and the whole hypothalami were isolated on ice and snap-frozen with liquid nitrogen. For acute cold exposure, C57Bl/6J mice were exposed to 4°C for 4 h before brains were taken out and different brain areas were isolated on ice using the mouse matrix (#RBM-2000C, ASI Instruments). Microdissected brain areas were immediately frozen with liquid nitrogen and kept at −80°C until further analysis.

For overnight fasting, C57BL/6J mice were fasted overnight for ∼16 h by removing food pellets from the food hoppers and changing the cage bottoms to clean ones. For the fed condition, cage bottoms were changed without removing food pellets. Water was freely available in both conditions. In the next morning, brains were harvested and different areas were isolated as described above.

For 2DG injection, C57BL/6J mice were injected with 600 mg/kg 2DG in saline (IP) and brains were harvested 4 h later and different areas were isolated as described above. Control mice were injected with saline (IP).

For the testing of glucocorticoid receptor (GR)-mediated *Zbtb16* induction, C57BL/6J mice were injected with 10 mg/kg dexamethasone (Dex, #1126, Tocris) in 1% ethanol in PBS (IP) for 4 h before brains were harvested and different areas were isolated as described above. Control mice were injected with 1% ethanol in PBS (IP). To test how the inhibition of GR affects *Zbtb16* induction by fasting, C57BL/6J mice were fasted overnight and vehicle (DMSO), 10 mg/kg or 50 mg/kg RU486/mifepristone (#1479, Tocris) in DMSO (IP) was injected. Brains were harvested 5 h later and different areas were isolated as described above.

### Immunohistochemistry and Histological Analysis

Mice were deeply anesthetized by isoflurane and brains were harvested by transcardial perfusion with 10% formalin. Brains were postfixed in 10% formalin at 4°C overnight and cryoprotected in 30% sucrose. Brains were sliced at 30 μm thickness into 4 series with a sliding microtome and processed to free-floating immunohistochemistry (IHC). Primary antibodies used in this study are chicken anti-GFP (ab13970, Abcam; 1:1000, RRID: AB_300798), rabbit anti-Trh (Dr. Eduardo A. Nillni, Brown University; 1:1000, EAN: pYE26), rabbit anti-Crh (T-4414.0050, Peninsula Laboratories, 1:1000, RRID: AB_518268), rabbit anti-Oxt (20068, Immunostar, 1:2000, RRID: AB_572258), rabbit anti-Avp (20069, Immunostar, 1:2000, RRID: AB_572219), rabbit anti-Nos1 (61–7000, Thermo Fisher Scientific, 1:500, RRID: AB_2313734), rabbit anti-Pomc (G-029-30, Phoenix Pharmaceuticals Inc., 1:500, RRID: AB_2617186), and rabbit anti-Zbtb16 (HPA-001499, Millipore Sigma, 1:500, RRID: AB_1079640). Secondary antibodies used in this study are donkey anti-chicken IgY Alexa Fluor 488 (703-546-155, Jackson ImmunoResearch Laboratories; 1:200, RRID: AB_2340376) and donkey anti-rabbit Alexa Fluor 594 (A-21207, Thermo Fisher Scientific; 1:200, RRID: AB_141637).

### Viruses and Stereotaxic Surgeries

Stereotaxic injection of adeno-associated virus (AAV) was performed as previously described (Rezai-Zadeh et al., 2014; Yu et al., 2018). Briefly, mice were placed on a stereotaxic alignment system (#1900, David Kopf Instruments) and maintained anesthetized by 1–2% isoflurane during surgeries. For *Zbtb16* KD in the PVH or the ARC, AAV9-U6/H1-Zbtb16 siRNA-GFP (AAV-Zbtb16 siRNA, 1.0 × 10^12^ vg/ml, Applied Biological Materials Inc., Richmond, BC, Canada) or AAV9-U6/H1-scrambled siRNA-GFP (AAV-Scrambled siRNA, 1.0 × 10^12^ vg/ml, Applied Biological Materials Inc., Richmond, BC, Canada) was injected bilaterally at 200 nl per site (400 nl total per animal). Virus was injected with a guide cannula and injection set (Plastics One) at 20 nl/30 s and the injection assembly was left in place for 5 min after the injection before removal and the skull and incision were closed with bone wax (Lukens, #901, Medline Industries) and wound clip (#203–1000, CellPoint Scientific). The coordinate for the PVH injection was AP: −0.6 mm, ML: ±0.4 mm, DV: −4.8 mm from the bregma, and for the ARC injection was AP: −1.45 mm, ML: ±0.3 mm, DV: −5.8 mm from the bregma.

For chemogenetic stimulation of Zbtb16 neurons in the PVH or the ARC, we injected AAV5-hSyn-DIO-hM3Dq-mCherry (AAV-DREADD-Gq, 3.8 × 10^12^ vg/ml, University of North Carolina Vector Core) or AAV5-hSyn-DIO-mCherry (AAV-Control, 5.2 × 10^12^ vg/ml, University of North Carolina Vector Core) bilaterally at 150 nl per site (300 nl total per animal) in *Zbtb16-Cre* mice with the same coordinates described above for *Zbtb16* KD.

### Knockdown of Zbtb16 in the PVH and the ARC

C57BL/6J mice were injected with either AAV-Scrambled siRNA or AAV-Zbtb16 siRNA in the PVH or the ARC at 2–4 months of age. The DNA sequence for scrambled siRNA is 5′-GGG TGA ACT CAC GTC AGA A-3′. For *Zbtb16* KD, 4 pooled siRNAs were expressed and their sequences are 5′-TGA GAT CCT CTT CCA CCG AAA CAG CCA GC-3′, 5′-CAT CTT TAT CTC GAA GCA TTC CAG CGA GG-3′, 5′-GTG GAC AGC TTG ATG AGT ATA GGA CAG TC-3′, 5′-AGT GCC AGA GAG CTG CAT TAT GGG AGA GA-3′. Approximately 3 weeks after virus injection, mice were housed in the PhenoMaster indirect calorimetry system (TSE systems) to measure energy expenditure, food intake, locomotor activity, and respiratory exchange ratio. Body composition was measured at the beginning of indirect calorimetry with a Minispec LF110 NMR analyzer (Bruker Biospin) and energy expenditure was normalized with lean body mass because we did not observe body composition difference between groups. Mice were housed at 23°C and acclimated for 3 days before being subject to manipulations. For acute cold exposure, chamber temperature was lowered to 10°C at 9 am and raised back to 23°C at 4 pm (total 7 h). For overnight fasting, food was removed and cage bottoms were replaced with clean ones at 5 pm, and food was provided back at 9 am the next morning (total 16 h).

Intraperitoneal glucose tolerance test (IPGTT) was conducted by fasting mice overnight, injecting glucose in the next morning (2 mg/g, IP), and measuring blood glucose level at 0 min and every 30 min for 2 h. A small amount of blood was taken from the tail vein and blood glucose was measured with glucose strips and a glucometer (OneTouch Ultra Strips and OneTouch Ultra glucometer, LifeScan Inc).

### Validation of Zbtb16 siRNA in Mouse Brown Adipocytes

An immortalized brown preadipocyte cell line (Uldry et al., 2006) was cultured and induced for differentiation as described previously (Chang et al., 2010). Briefly, preadipocytes were grown to confluence in DMEM culture medium supplemented with 20 nM insulin and 1 nM T3 (differentiation medium) on a 48-well plate. Differentiation was then induced by incubating the cells in differentiation medium supplemented with 0.5 mM isobutylmethylxanthine, 0.5 μM Dex, and 0.125 mM indomethacin for 48 h. Thereafter, the cells were maintained in differentiation medium until day 7. For AAV-mediated silencing of *Zbtb16* expression, differentiated brown adipocytes were infected with AAV expressing scrambled (control) or *Zbtb16* siRNA for 16 h and *Zbtb16* mRNA expression was analyzed 72 h after infection. AAVs were infected at two different titers, 10^4^ MOI and 10^5^ MOI. For the induction of *Zbtb16* gene expression, differentiated brown adipocytes were serum-starved for 8 h and treated with vehicle or dexamethasone (10 μM) for 5 h (from hour 3 to hour 8) before the collection of mRNA.

### Chemogenetic Stimulation of Zbtb16 Neurons in the PVH and the ARC

We injected either AAV-DREADD-Gq or AAV-Control into the PVH or the ARC of *Zbtb16-Cre* mice (Constantinides et al., 2014) at 2–4 months of age as described above. Mice were used for experiments 3 weeks after the virus injection. Mice with the PVH virus injection were injected with clozapine-N-oxide (CNO, C0832, Millipore Sigma) at 1 mg/kg, IP and rectal temperature was measure every 20 min for 2 h with a micro thermometer (227-193, ThermoWorks). Mice with the ARC virus injection were injected with either PBS or CNO (1 mg/kg, IP) in the morning and food weight was measured at time 0, 2, and 4 h to calculate 2 and 4 h food intake. 10 days later, the injection was reversed and the food intake was measured again in the same mice to compare PBS vs. CNO. 4 h food intake after CNO injection was compared to the amount of food consumed for 4 h after overnight fasting in the same mice. Both PVH- and ARC-injected mice were housed in Comprehensive Laboratory Animal Monitoring System (CLAMS; Columbus Instruments) to measure energy expenditure, locomotor activity, and RER upon chemogenetic stimulation of Zbtb16 neurons in the PVH or the ARC. CNO was injected at 1.0 mg/kg, IP in the morning and 2 h data were averaged for each parameter. Each mouse was administered with PBS and CNO, and the injection order was randomly assigned and counterbalanced.

### Statistical Analysis

**FIGURE 1:**
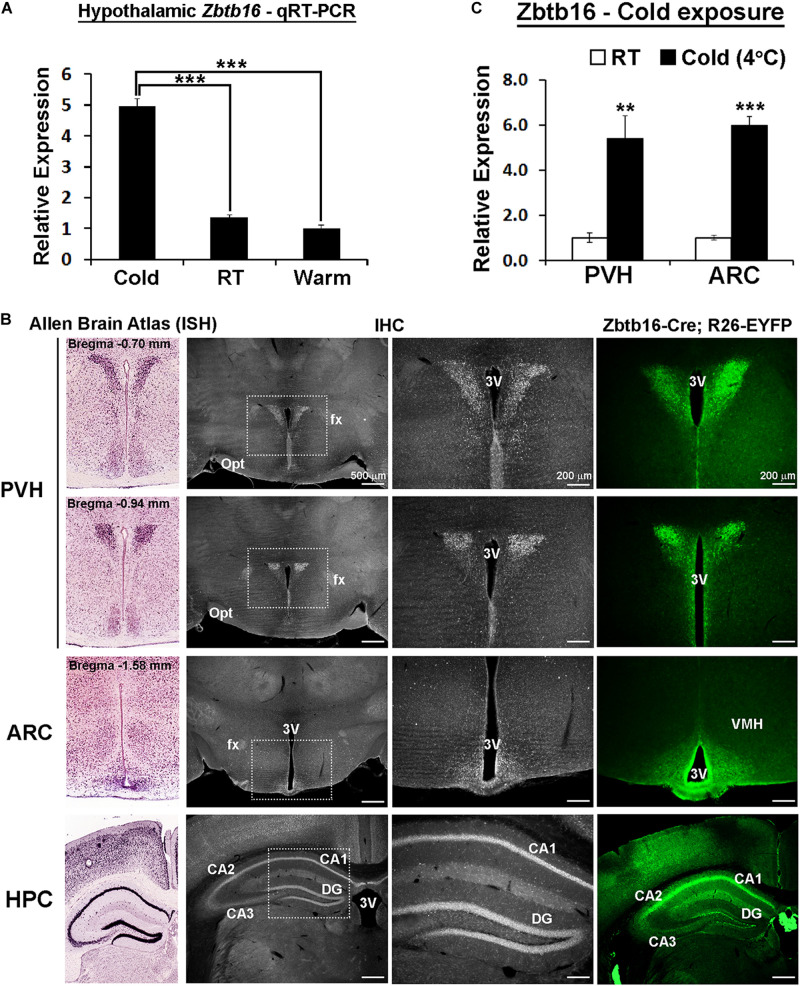
Zbtb16 expression is induced by cold exposure in the hypothalamic paraventricular and arcuate nuclei. **(A)** *Zbtb16* mRNA expression is induced in the mouse hypothalamus by 3 h of cold exposure (Cold at 4°C, *n* = 4; RT at 22°C, *n* = 4; Warm at 35°C, *n* = 4; one-way ANOVA). **(B)** mRNA expression (from Allen Brain Atlas, far left column, experiment #71717125), protein expression (IHC, two middle columns), and reporter expression (EYFP expression driven by *Zbtb16-Cre*, far right column) show consistent expression in the PVH, ARC, and hippocampus (HPC). **(C)** *Zbtb16* mRNA expression is increased by 4 h of cold exposure in both the PVH and the ARC (4°C, *n* = 5; RT, *n* = 5; independent *t*-test in each area). Data were represented by mean ± SEM. **p < 0.01, ***p < 0.001. 3V, 3rd ventricle; CA1, field CA1 hippocampus; CA2, field CA2 hippocampus; CA3, field CA3 hippocampus; DG, dentate gyrus; fx, fornix; Opt, optic tract; VMH, ventromedial hypothalamic nucleus.

**FIGURE 2:**
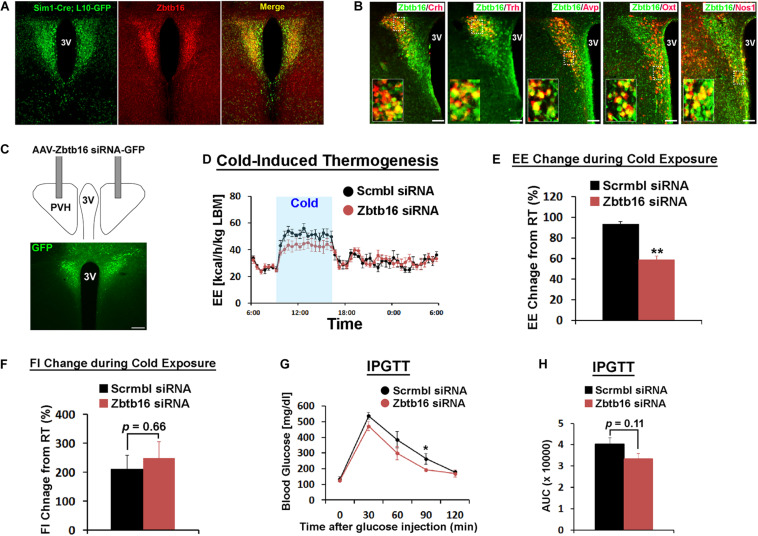
Zbtb16 in the PVH contributes to cold-induced thermogenesis and glycemic control. **(A)** Zbtb16 protein expression (IHC, red) was compared to Sim1 expression (Sim1-Cre; L10-GFP reporter, green) in the PVH. **(B)** Zbtb16 is expressed in multiple cell types in the PVH including neurons expressing Crh, Trh, Avp, Oxt, and Nos1. Scale bar is 200 μm. **(C)** Schematic diagram showing bilateral injection of AAV expressing siRNA against *Zbtb16* in the PVH and a representative histological image showing correct targeting of the virus, shown by GFP expression. Scale bar is 200 μm. **(D)** Zbtb16^PVH^ KD mice (red, *n* = 6) exhibited attenuated cold (10°C)-induced thermogenic response compared to mice injected with AAV expressing scrambled siRNA (black, *n* = 4). 24 h energy expenditure data were analyzed by repeated measures ANOVA followed by Bonferroni pairwise comparisons. **(E)** Average energy expenditure during cold exposure (7 h from 9 am to 4 pm) was significantly lower in Zbtb16^PVH^ KD mice (red, *n* = 6) compared to control mice (black, *n* = 4). Data were analyzed by independent *t*-test. **(F)** Food intake during cold exposure was not different between groups (scrambled siRNA, *n* = 4; Zbtb16 siRNA, *n* = 6; independent *t*-test). **(G)** Intraperitoneal glucose tolerance test (IPGTT) revealed slightly improved glucose tolerance in Zbtb16^PVH^ KD mice (red, *n* = 6) compared to control mice (black, *n* = 4). Data were analyzed by repeated measures ANOVA followed by Bonferroni pairwise comparisons. **(H)** Area under the curve comparison for the IPGTT data shown in **(G)** (scrambled siRNA, *n* = 4; Zbtb16 siRNA, *n* = 6; independent *t*-test). Data were represented by mean ± SEM, *p < 0.05, **p < 0.01.

**FIGURE 3:**
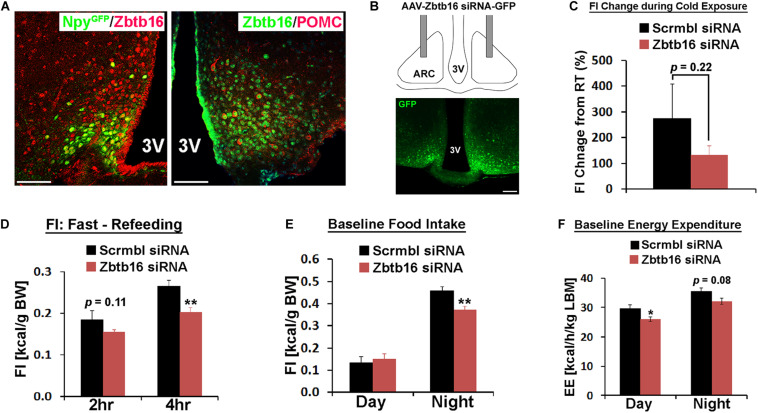
Zbtb16 in the ARC contributes to food intake control. **(A)** Representative histological images showing co-expression of Zbtb16 with Npy-GFP or Pomc in the ARC. Scale bar is 50 μm. **(B)** Schematic diagram showing bilateral injection of AAV expressing siRNA against *Zbtb16* in the ARC and a representative histological image showing correct targeting of the virus, shown by GFP expression. **(C)** Zbtb16^ARC^ KD mice (red, *n* = 7) showed reduced food intake during cold exposure (10°C for 7 h from 9 am to 4 pm) compared to mice injected with AAV expressing scrambled siRNA (black, *n* = 4). Data were analyzed by independent *t*-test. **(D)** Zbtb16^ARC^ KD mice exhibited reduced food intake after overnight fasting (scrambled siRNA, *n* = 4; Zbtb16 siRNA, *n* = 7; repeated measures ANOVA followed by Bonferroni pairwise comparisons). **(E)** Zbtb16^ARC^ KD mice showed decreased nighttime food intake at baseline (scrambled siRNA, *n* = 4; Zbtb16 siRNA, *n* = 7; repeated measures ANOVA followed by Bonferroni pairwise comparisons). **(F)** Zbtb16^ARC^ KD mice (red, *n* = 7) showed decreased baseline energy expenditure compared to control mice (black, *n* = 4). Data were analyzed by repeated measures ANOVA followed by Bonferroni pairwise comparisons. Data were represented by mean ± SEM, *p < 0.05, **p < 0.01.

**FIGURE 4:**
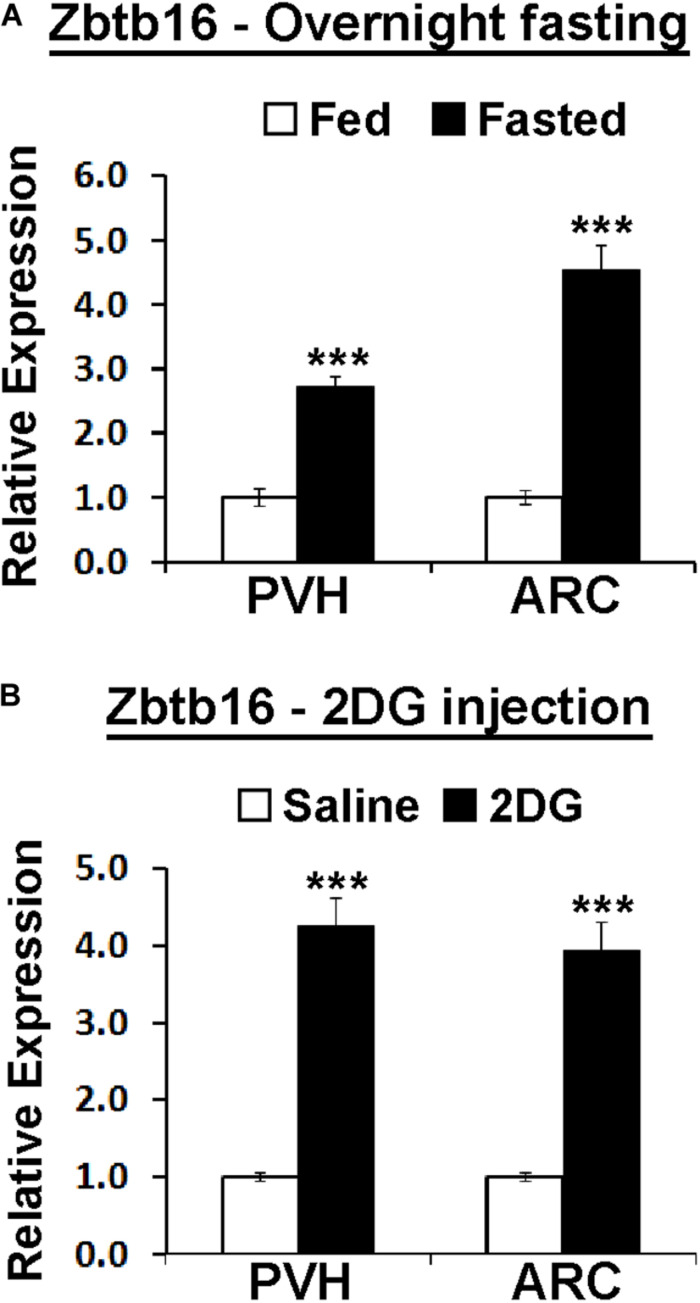
Hypothalamic Zbtb16 is induced by energy deficit. **(A)** Zbtb16 is induced by overnight fasting in both the PVH and the ARC (fed, *n* = 5; fasted, *n* = 6; independent *t*-test in each area). **(B)** Glucopenia induced by IP injection of 2DG (600 mg/kg) induced *Zbtb16* in both the PVH and the ARC (saline, *n* = 5; 2DG, *n* = 5; independent *t*-test in each area). Tissues were harvested 4 h after the 2DG injection. Data were represented by mean ± SEM, ***p < 0.001.

**FIGURE 5:**
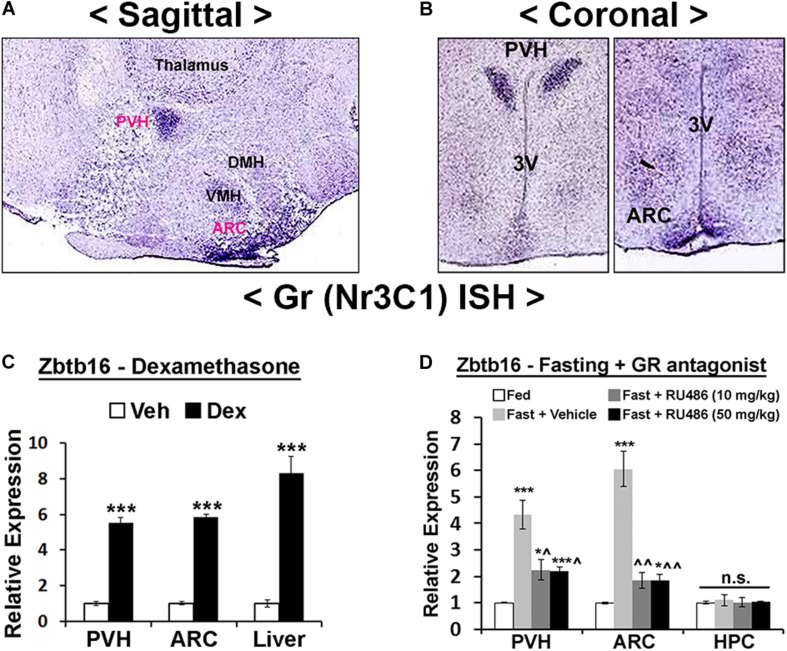
Hypothalamic Zbtb16 is induced by glucocorticoid receptor signaling. **(A,B)** GR mRNA expression in the mouse hypothalamus shows its enrichment in the PVH and ARC (Allen Brain Atlas, experiment #727 and #728). **(C)** IP injection of GR agonist Dex (10 mg/kg) induced *Zbtb16* in the PVH, ARC, and liver (vehicle, *n* = 6; Dex, *n* = 6; independent *t*-test). **(D)** *Zbtb16* induction by overnight fasting was significantly attenuated by GR antagonist RU486/Mifepristone (IP at 10 mg/kg or 50 mg/kg for 5 h) in the PVH and ARC. Neither fasting nor RU486 affected *Zbtb16* expression in the hippocampus (HPC) (*n* = 5 for each condition; one-way ANOVA in each area). Data are represented by mean ± SEM. *p < 0.05, ***p < 0.001. ^*p* < 0.05, ^^*p* < 0.01 compared to the “Fast + Vehicle” condition in **(D)**.

**FIGURE 6:**
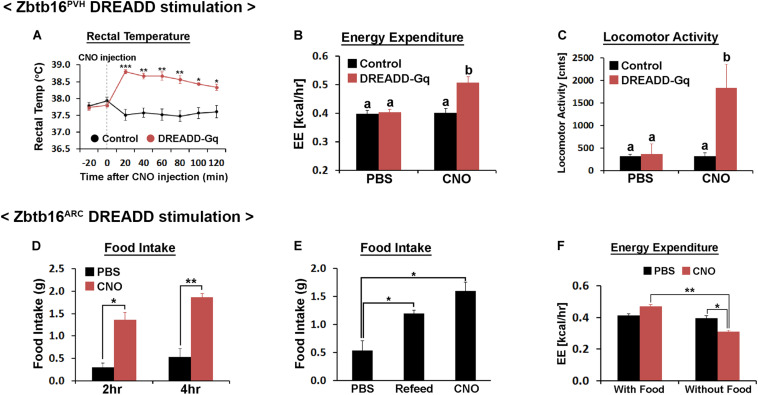
Chemogenetic stimulation of Zbtb16^PVH^ or Zbtb16^ARC^ neurons. **(A)** Chemogenetic stimulation of Zbtb16^PVH^ neurons increased rectal temperature in mice (control, *n* = 9; DREADD-Gq, *n* = 3; repeated measures ANOVA followed by Bonferroni pairwise comparisons). **(B)** Chemogenetic stimulation of Zbtb16^PVH^ neurons increased 2 h energy expenditure in mice after CNO injection (control, *n* = 9; DREADD-Gq, *n* = 3; repeated measures ANOVA followed by Bonferroni pairwise comparisons). Bars with different letters denote statistical significance at *p* < 0.01. **(C)** Chemogenetic stimulation of Zbtb16^PVH^ neurons increased 2 h locomotor activity in mice after injection (control, *n* = 9; DREADD-Gq, *n* = 3; repeated measures ANOVA followed by Bonferroni pairwise comparisons). Bars with different letters denote statistical significance at *p* < 0.01. **(D)** Chemogenetic stimulation of Zbtb16^ARC^ neurons increased daytime food intake measured at 2 and 4 h after injection (*n* = 4; repeated measures ANOVA followed by Bonferroni pairwise comparisons). **(E)** Chemogenetic stimulation of Zbtb16^ARC^ neurons increased 4 h food intake during daytime which is comparable to the amount of food eaten after overnight fasting in the same mice (*n* = 4; repeated measures ANOVA followed by Bonferroni pairwise comparisons). **(F)** Chemogenetic stimulation of Zbtb16^ARC^ neurons decreased 2 h energy expenditure only when food was absent (*n* = 4, repeated measures ANOVA followed by Bonferroni pairwise comparisons). CNO was injected at 1 mg/kg, IP in all experiments. Data are represented by mean ± SEM, *p < 0.05, **p < 0.01, ***p < 0.001.

Data are represented as mean ± SEM. All statistical analyses were done with SPSS 24 (IBM) and *p* < 0.05 was considered statistically significant. In all graphs, *p < 0.05, **p < 0.01, and ***p < 0.001. Some bar graphs used letters to indicate statistical significance between comparisons. For more detailed information, see section “Results” and Figures 1–6.

## Results

### Zbtb16 Expression Is Induced by Cold Exposure in the Hypothalamic Paraventricular and Arcuate Nuclei

*Zbtb16* was first identified from our previous genetic screen in the mouse hypothalamus as a gene upregulated by 3 h of cold exposure (Figure 1A and unpublished data). More detailed expression analysis revealed that *Zbtb16* is expressed in the PVH and the ARC in the hypothalamus even though it shows the strongest expression in the hippocampus in the brain (Figure 1B). We did not detect any significant Zbtb16 expression in other hypothalamic areas. *Zbtb16* mRNA expression was induced by acute cold exposure in both the PVH and the ARC to a similar degree when its expression was analyzed with microdissected brain tissues (Figure 1C).

### Zbbtb16 in the PVH Contributes to Cold-Induced Thermogenesis and Glycemic Control

Because of the expression of *Zbtb16* in two nuclei that play critical roles in energy homeostasis and its induction by cold exposure, we carried out a series of experiments to figure out its role in each nucleus. Sim1 is a transcription factor that marks PVH neurons (Balthasar et al., 2005), and the majority of Zbtb16 neurons in the PVH co-expressed *Sim1-Cre*-driven GFP reporter even though *Zbtb16* expression is restricted to the anterior half of the PVH (Figure 2A). The PVH harbors heterogeneous neuronal populations that are involved in numerous homeostatic functions and marked by different gene expressions. Double IHC of Zbtb16 with several of these markers (i.e., Crh, Trh, Avp, Oxt, and Nos1) revealed significant co-expression of Zbtb16 with all marker genes tested (Figure 2B, 95.3 ± 0.01% of Crh, 93.0 ± 0.02 % of Trh, 92.3 ± 0.01% of Avp, 94.7 ± 0.01% of Oxt, and 88.0 ± 0.02% of Nos1 neurons co-express Zbtb16; *n* = 3 for each marker).

To test the relevance of Zbtb16 in whole body physiology, we injected AAV expressing a pool of siRNAs against *Zbtb16* (AAV-Zbtb16 siRNA) bilaterally into the PVH in mice (Figure 2C). *Zbtb16* expression was shown to be induced in brown adipocytes by the GR agonist Dex (Chen et al., 2014), and AAV-Zbtb16 siRNA attenuated this induction by around 50% (Supplementary Figures 1A,B). We first tested how *Zbtb16* KD in the PVH (Zbtb16^PVH^ KD) affected physiological responses to cold exposure. While there was no change in energy expenditure between mice injected with AAV-Zbtb16 siRNA and mice injected with AAV-scrambled siRNA at room temperature (RT, 22°C; Supplementary Figure 2A), Zbtb16^PVH^ KD mice showed reduced capacity to increase their energy expenditure during cold exposure (Figures 2D,E). Because multiple neuronal populations in the PVH contribute to the regulation of feeding behavior (Balthasar et al., 2005; Krashes et al., 2014; Pei et al., 2014; Sutton et al., 2014), we also tested whether Zbtb16^PVH^ plays a role in controlling feeding behavior. However, hyperphagic response during cold exposure and diurnal food intake were not affected by Zbtb16^PVH^ KD (Figure 2F and Supplementary Figures 2A,B). IPGTT showed a slightly improved glycemic control in Zbtb16^PVH^ KD mice (Figure 2G) even though the area under the curve comparison did not reach statistical significance (Figure 2H).

### Zbtb16 in the ARC Contributes to Food Intake Control

In the ARC, two counteracting neuronal populations play a critical role in food intake regulation and overall energy homeostasis. Consistent with previous single-cell transcriptome analyses (Henry et al., 2015; Campbell et al., 2017), *Zbtb16* is expressed in both orexigenic Agrp/Npy neurons and anorexigenic Pomc neurons even though not all Pomc neurons co-expressed *Zbtb16* while all Agrp/Npy neurons co-expressed *Zbtb16* (Figure 3A, cell counting not shown). Similar to the PVH, we bilaterally injected AAV-Zbtb16 siRNA into the ARC in mice and investigated the role of Zbtb16 in this nucleus (Figure 3B). While thermogenic response to cold was not affected in Zbtb16^ARC^ KD mice (Supplementary Figure 3A), the amount of food consumed during cold exposure was reduced in these mice even though it did not reach the statistical significance (Figure 3C). Furthermore, Zbtb16^ARC^ KD mice ate less during the refeeding after overnight fasting and the dark cycle (Figures 3D,E). Interestingly, baseline energy expenditure was slightly lower in Zbtb16^ARC^ KD mice compared to control mice (Figure 3F) even though hypometabolic response to fasting was not different between groups (Supplementary Figure 3B). The lower metabolic rate in Zbtb16^ARC^ KD mice could be an adaptive response to reduced feeding as the body weight of these mice was not different from control mice (data not shown).

### Zbtb16 Expression Is Induced by Energy Deficit in the PVH and the ARC via Glucocorticoid Receptor Signaling

Because of the effect of Zbtb16^ARC^ KD on food intake, we investigated whether the negative energy balance created by overnight fasting can induce *Zbtb16* expression similarly to cold exposure. Indeed, overnight fasting in mice induced *Zbtb16* expression in both the PVH and the ARC (Figure 4A). *Zbtb16* induction by overnight fasting prompted us to test whether a different energy deficit signal can induce *Zbtb16* expression. 2DG is a glucoprivic agent that blocks glycolysis and triggers strong counter-regulatory responses to elevate blood glucose levels (Ritter et al., 2001). The injection of 2DG (600 mg/kg, IP) robustly induced *Zbtb16* expression in both the PVH and the ARC (Figure 4B).

We next probed the upstream mechanisms responsible for *Zbtb16* induction. Cold exposure, overnight fasting, and 2DG injection all induce an energy deficit state that triggers the release of GCs from the adrenal gland (Wilkerson et al., 1974; Yi and Baram, 1994; Makimura et al., 2003; Magomedova and Cummins, 2016). Furthermore, previous studies showed induction of *Zbtb16* in several non-neuronal cells by GCs (Fahnenstich et al., 2003; Chen et al., 2014; Naito et al., 2015). In the hypothalamus, GR shows a similarly enriched expression pattern in the PVH and the ARC as *Zbtb16* (Figures 5A,B), and the injection of the GR agonist Dex in mice robustly induced *Zbtb16* in the PVH and the ARC (Figure 5C). The induction of *Zbtb16* by overnight fasting was strongly inhibited by the injection of the GR antagonist RU486/Mifepristone (Figure 5D). Interestingly, *Zbtb16* was neither induced by overnight fasting nor suppressed by RU486 in the hippocampus, implying a different transcriptional regulation of *Zbtb16* in this area (Figure 5D).

### Chemogenetic Stimulation of Zbtb16 Neurons

While the intracellular mechanisms downstream of Zbtb16 is currently elusive, we reasoned that they are likely to involve the modulation of neuronal activity. Therefore, we hypothesized that chemogenetic stimulation of Zbtb16^PVH^ neurons would result in a change in energy expenditure while that of Zbtb16^ARC^ neurons would affect food intake. We injected AAV-DREADD-Gq bilaterally into *Zbtb16-Cre* mice in the PVH or the ARC for a chemogenetic stimulation of Zbtb16 neurons in each area (Supplementary Figure 4).

Injection of CNO (1 mg/kg, IP) in Zbtb16^PVH^ DREADD-Gq mice increased core temperature, which lasted at least for 2 h (Figure 6A). Consistent with this phenotype, energy expenditure during the same period increased about 25% in Zbtb16^PVH^ DREADD-Gq mice with CNO injections (Figure 6B). Locomotor activity during the same span also increased in Zbtb16^PVH^ DREADD-Gq mice with CNO (Figure 6C), consistent with the fact that Zbtb16 is expressed in Crh neurons in the PVH (Fuzesi et al., 2016). Food intake was not affected by chemogenetic stimulation of Zbtb16^PVH^ neurons (data not shown), corroborating findings from the Zbtb16^PVH^ KD study.

Chemogenetic stimulation of Zbtb16^ARC^ neurons increased daytime food intake in fed mice, which was comparable to the amount of food consumed after overnight fasting in the same mice (Figures 6D,E). Accordingly, locomotor activity in Zbtb16^ARC^ DREADD-Gq mice increased sharply with CNO injection in the absence of food, reflecting food-seeking behavior. These phenotypes are reminiscent of the stimulation of Agrp/Npy neurons (Aponte et al., 2011; Krashes et al., 2011). Energy expenditure after CNO injection decreased in Zbtb16^ARC^ DREADD-Gq mice when food was not provided (Figure 6F), again consistent with what was observed with chemogenetic stimulation of Agrp/Npy neurons (Krashes et al., 2011). Rectal temperature was not affected when Zbtb16_ARC_ neurons were stimulated (data not shown).

## Discussion

In this study, we describe for the first time the expression and potential role of the newly identified transcription factor Zbtb16 in the hypothalamus. We uncovers that *Zbtb16* expression is induced by various conditions of energy deficit in the PVH and the ARC through GC signaling. This induction contributes to modulating energy expenditure, food intake, and glycemic control to properly respond to the energy deficit state. Chemogenetic stimulation of Zbtb16 neurons in the PVH and the ARC modulates energy expenditure and food intake, respectively, consistent with results from *Zbtb16* KD in each nucleus, suggesting that *Zbtb16* induction is likely to affect neuronal activity. However, many unanswered questions remain.

The most important unanswered question would be how Zbtb16 affects physiology and behavior. As a transcription factor, Zbtb16 could regulate the expression of neuropeptides, ion channels, or various receptors to influence the response to incoming signals and/or the signaling to downstream neurons. *Zbtb16* is expressed in various tissues and carries out diverse biological functions through regulation of tissue-specific target genes (Barna et al., 2000; Kovalovsky et al., 2008; Savage et al., 2008; Hobbs et al., 2010; Plaisier et al., 2012; Chen et al., 2014; Liska et al., 2017), making it difficult to postulate its mechanism in the hypothalamus. The fact that *Zbtb16* is expressed in heterogeneous neuronal populations makes the issue more complicated because it is currently not clear whether *Zbtb16* is equally induced by energy deficit in all neurons it is expressed or in a specific subpopulation, and whether it regulates the same genes in different neurons. Studies with selective ablation of *Zbtb16* in different neuronal populations would greatly enhance our understanding of where and how Zbtb16 functions. Nevertheless, our findings provide important clues on how Zbtb16 functions in the hypothalamus. For example in the PVH, several neuronal populations (e.g., Mc4r, Pdyn, Trh, Pacap, and Nos1 neurons) have been shown to control feeding behavior (Krashes et al., 2014; Sutton et al., 2014; Li et al., 2019) but neither Zbtb16^PVH^ KD nor Zbtb16^PVH^ DREADD-Gq affected food intake, implying that Zbtb16 probably does not affect functions of these neurons. On the other hand, PVH Oxt neurons were shown to increase energy expenditure without affecting food intake through their projection to the spinal cord (Sutton et al., 2014), indicating a potential role of Zbtb16 in these neurons. Zbtb16 may also affects energy expenditure secondarily through the release of Trh as Trh was shown to increase energy expenditure (Mullur et al., 2014). Interestingly, Zbtb16^PVH^ KD only affects cold-induced thermogenesis but not fasting-evoked hypometabolism even though the *Zbtb16* levels elevate in both circumstances. This result indicates that Zbtb16 functions in a context-dependent manner and the *Zbtb16* induction by energy deficit in the PVH is not related to energy expenditure. We reason that the major function of Zbtb16^PVH^ during energy deficit is to control blood glucose levels even though Zbtb16^PVH^ KD only mildly improved glucose tolerance.

In the ARC, Zbtb16^ARC^ KD decreases food intake while chemogenetic stimulation of Zbtb16^ARC^ neurons increases food intake, in line with *Zbtb16* expression in AgRP/Npy neurons. Although *Zbtb16* is also expressed in POMC neurons, previous single cell analyses showed that *Zbtb16* is expressed at a higher level and induced more robustly by fasting in AgRP/Npy neurons (Henry et al., 2015; Campbell et al., 2017). Therefore, we speculate that the main function of Zbtb16 in the ARC is to promote feeding in the face of energy deficit downstream of GC signaling in Agrp/Npy neurons. This idea is congruent with previous studies that showed increased food intake by central GR stimulation (Debons et al., 1986; Zakrzewska et al., 1999; Cusin et al., 2001; Veyrat-Durebex et al., 2012). Slightly reduced energy expenditure in Zbtb16^ARC^ KD mice may be an adaptive response to reduced food intake as we did not observe altered energy expenditure during cold exposure or fasting in the same mice. Nevertheless, the effect of Zbtb16^ARC^ on energy expenditure will have to be investigated more carefully in future studies.

The fact that *Zbtb16* expression is induced by GC signaling bears significant implications in Zbtb16’s involvement in energy homeostasis. In addition to their function in the periphery, GCs centrally control feeding behavior, energy expenditure, and autonomic output to peripheral organs (Zakrzewska et al., 1999; Cusin et al., 2001; Kellendonk et al., 2002; Bernal-Mizrachi et al., 2007; Veyrat-Durebex et al., 2012; Yi et al., 2012; Laryea et al., 2013; Solomon et al., 2015; Perry et al., 2019). More importantly, GCs are required for the development of both genetic and diet-induced obesity and interact with leptin signaling (Yukimura and Bray, 1978; Saito and Bray, 1984; Bray et al., 1992; Makimura et al., 2000; Perry et al., 2019). Therefore, central Zbtb16 may contribute to the control of energy balance and ultimately body weight downstream of GC signaling. However, we cannot rule out the regulation of *Zbtb16* expression by other hormones or signaling molecules, as RU486 incompletely blocks the *Zbtb16* induction by fasting. Factors released during energy deficit (e.g., ghrelin) or surplus (e.g., leptin, insulin) might very well be involved in the induction or suppression of *Zbtb16* in the hypothalamus, respectively.

Several rare single nucleotide polymorphisms in the *Zbtb16* locus have been associated with higher body mass index, waist to hip ratio, and LDL cholesterol levels in humans (Bendlova et al., 2017), and losing one copy of *Zbtb16* caused reduced body weight and adiposity in rats (Liska et al., 2017) and mice (our own unpublished data). It is particularly interesting to investigate if Zbtb16 plays a role in weight regain after weight loss because the weight regain driven by enhanced appetite and reduced metabolic rate is a critical barrier for the long-term success of weight loss (Rosenbaum and Leibel, 2014).

## Data Availability Statement

The original contributions presented in the study are included in the article/Supplementary Material, further inquiries can be directed to the corresponding author/s.

## Ethics Statement

The animal study was reviewed and approved by the Institutional Animal Care and Use Committee at Pennington Biomedical Research Center.

## Author Contributions

SY conceptualized and planned the study. SY, HC, SP, and JL executed the experiments and analyzed the data. SY and JC wrote the manuscript. All authors contributed to the article and approved the submitted version.

## Conflict of Interest

The authors declare that the research was conducted in the absence of any commercial or financial relationships that could be construed as a potential conflict of interest.

## Supplementary Material

Supplementary Figure 1 Zbtb16 knockdown validation by AAV-mediated expression of Zbtb16 siRNA. **(A)** In cultured brown adipocytes, *Zbtb16* expression was highly induced by Dex treatment. Cells were serum-starved for 8 h and Dex was treated for 5 h (3 h into starvation). *n* = 4 for each condition (one-way ANOVA). **(B)** Dex (10 μM)-mediated *Zbtb16* induction in cultured brown adipocytes was significantly attenuated by AAV-mediated expression of Zbtb16 siRNA. Scrambled siRNA was transduced at 10^5^ MOI, Zbtb16 siRNA-Lo at 10^4^ MOI, and Zbtb16 siRNA-Hi at 10^5^ MOI (*n* = 2 for each condition; one-way ANOVA). Data are represented by mean ± SEM, ***p* < 0.01, ****p* < 0.001.

Supplementary Figure 2 Zbtb16 knockdown in the PVH. **(A,B)** Zbtb16^PVH^ KD affected neither energy expenditure nor food intake at baseline (scrambled siRNA, *n* = 4; Zbtb16 siRNA, *n* = 6; repeated measures ANOVA followed by Bonferroni pairwise comparisons). **(C)** Zbtb16^PVH^ KD did not affect hypometabolic response during fasting (scrambled siRNA, *n* = 4; Zbtb16 siRNA, *n* = 6; independent *t*-test). Data are represented by mean ± SEM.

Supplementary Figure 3 Zbtb16 knockdown in the ARC. **(A,B)** Zbtb16^ARC^ KD affected neither cold-adaptive thermogenesis nor fasting-induced hypometabolism (scrambled siRNA, *n* = 4; Zbtb16 siRNA, *n* = 7; independent *t*-test). **(C)** Zbtb16^ARC^ KD did not affect glucose tolerance (scrambled siRNA, *n* = 4; Zbtb16 siRNA, *n* = 7; repeated measures ANOVA followed by Bonferroni pairwise comparisons). Data are represented by mean ± SEM.

Supplementary Figure 4 Chemogenetic Stimulation of Zbtb16 neurons. **(A)** Representative histological images showing the expression of cFos (neuronal activation) and mCherry (virus) in the PVH in Zbtb16^PVH^ Control and DREADD-Gq mice. The brains were harvested 1 h after CNO injection at 1.0 mg/kg, IP. **(B)** Representative histological image showing the expression of cFos and mCherry in the ARC in Zbtb16^PVH^ DREADD-Gq mice. The brains were harvested 1 h after CNO injection at 1.0 mg/kg, IP.
