# Supplementary material for: Zinc Finger Transcription Factor Zbtb16 Coordinates the Response to Energy Deficit in the Mouse Hypothalamus
Source: Front Neurosci. 2020 Dec 1;14:592947. doi: 10.3389/fnins.2020.592947 (PMC7736175; doi:10.3389/fnins.2020.592947)
Supplement: Supplementary Figure 1 — Zbtb16 knockdown validation by AAV-mediated expression of Zbtb16 siRNA. (A) In cultured brown adipocytes, Zbtb16 expression was highly induced by Dex treatment. Cells were serum-starved for 8 h and Dex was treated for 5 h (3 h into starvation). n = 4 for each condition (one-way ANOVA). (B) Dex (10 μM)-mediated Zbtb16 induction in cultured brown adipocytes was significantly attenuated by AAV-mediated expression of Zbtb16 siRNA. Scrambled siRNA was transduced at 105 MOI, Zbtb16 siRNA-Lo at 104 MOI, and Zbtb16 siRNA-Hi at 105 MOI (n = 2 for each condition; one-way ANOVA). Data are represented by mean ± SEM, **p < 0.01, ***p < 0.001. [file Data_Sheet_2.PDF]

Supplementary Material

< Zbtb16 siRNA validation >

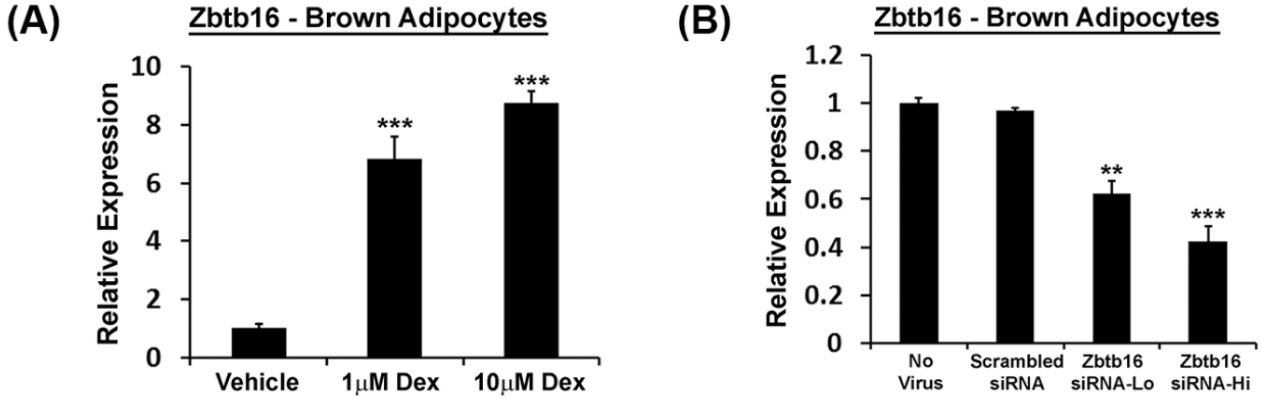

Supplementary Figure 1. Zbtb16 knockdown validation by AAV-mediated expression of Zbtb16 siRNA. (A) In cultured brown adipocytes, *Zbtb16* expression was highly induced by Dex treatment. Cells were serum-starved for 8 hrs and Dex was treated for 5 hrs (3 hrs into starvation). n=4 for each condition (one-way ANOVA). **(B)** Dex (10 μM)-mediated *Zbtb16* induction in cultured brown adipocytes was significantly attenuated by AAV-mediated expression of Zbtb16 siRNA. Scrambled siRNA was transduced at 10^5 MOI, Zbtb16 siRNA-Lo at 10^4 MOI, and Zbtb16 siRNA-Hi at 10^5 MOI (n=2 for each condition; one-way ANOVA). Data are represented by mean ± s.e.m. **$p < 0.01$, ***$p < 0.001$.

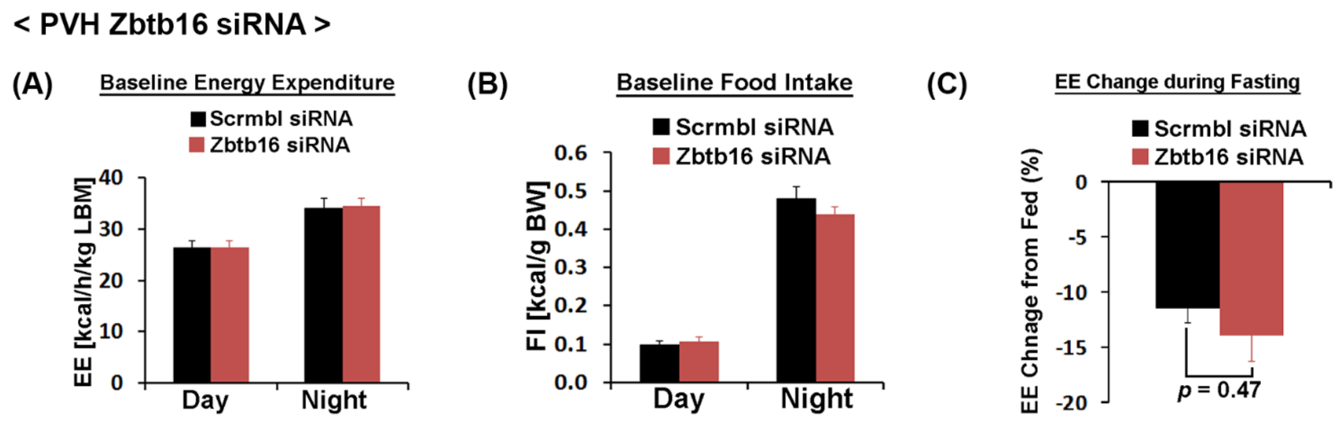

Supplementary Figure 2. Zbtb16 knockdown in the PVH. (A-B) Zbtb16^{PVH} KD affected neither energy expenditure nor food intake at baseline (scrambled siRNA, n=4; Zbtb16 siRNA, n=6; repeated measures ANOVA followed by Bonferroni pairwise comparisons). **(C)** Zbtb16^{PVH} KD did not affect hypometabolic response during fasting (scrambled siRNA, n=4; Zbtb16 siRNA, n=6; independent *t*-test). Data are represented by mean ± s.e.m.

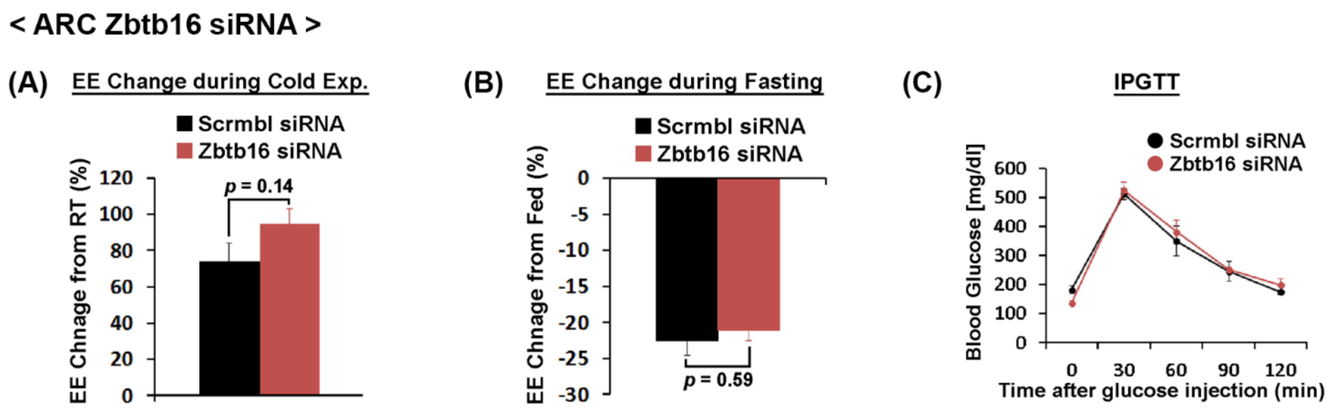

Supplementary Figure 3. Zbtb16 knockdown in the ARC. (A-B) Zbtb16^{ARC} KD affected neither cold-adaptive thermogenesis nor fasting-induced hypometabolism (scrambled siRNA, n=4; Zbtb16 siRNA, n=7; independent *t*-test). **(C)** Zbtb16^{ARC} KD did not affect glucose tolerance (scrambled siRNA, n=4; Zbtb16 siRNA, n=7; repeated measures ANOVA followed by Bonferroni pairwise comparisons). Data are represented by mean ± s.e.m.

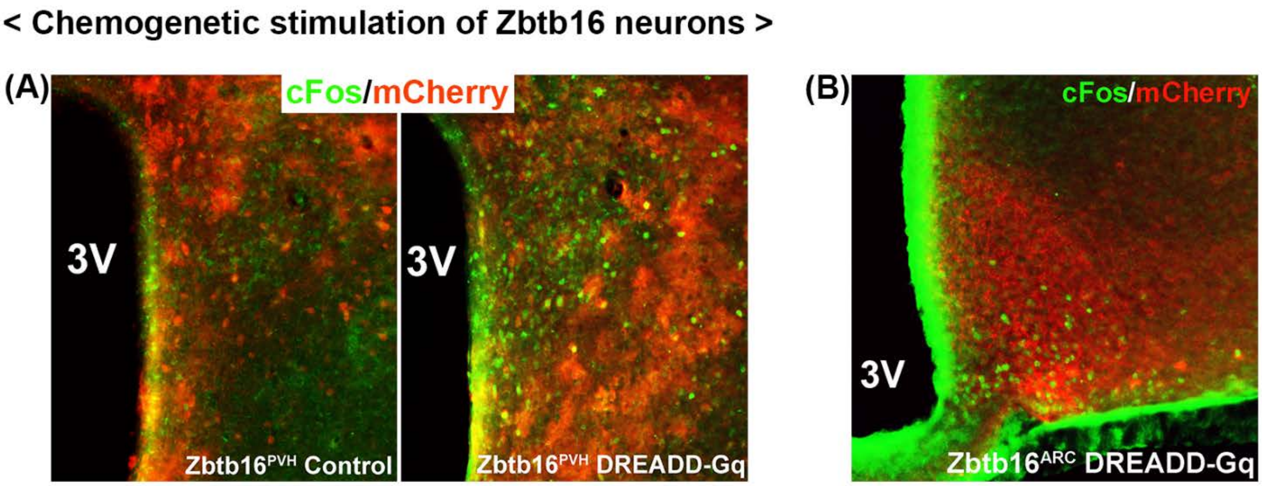

Supplementary Figure 4. Chemogenetic Stimulation of Zbtb16 neurons. (A) Representative histological images showing the expression of cFos (neuronal activation) and mCherry (virus) in the PVH in Zbtb16^{PVH} Control and DREADD-Gq mice. The brains were harvested 1hr after CNO injection at 1.0 mg/kg, IP. **(B)** Representative histological image showing the expression of cFos and mCherry in the ARC in Zbtb16^{PVH} DREADD-Gq mice. The brains were harvested 1hr after CNO injection at 1.0 mg/kg, IP.
